# Quantitative benefit-risk analysis for prophylactic vaccines in the context of FDAs benefit-risk framework

**DOI:** 10.1038/s41541-025-01342-3

**Published:** 2026-02-05

**Authors:** Hong Yang, Osman N. Yogurtcu, Ujwani Nukala, Patrick R. Funk, Hector S. Izurieta, Richard A. Forshee

**Affiliations:** 1 Office of Biostatistics and Pharmacovigilance, Center for Biologics Evaluation and Research, US FDA, Silver Spring, MD USA; 2 Office of Vaccines Research and Review, Center for Biologics Evaluation and Research, US FDA, Silver Spring, MD USA; 3 Office of Surveillance and Epidemiology, Center for Drug Evaluation and Research, US FDA, Silver Spring, MD USA

**Keywords:** Computational biology and bioinformatics, Drug discovery, Health care, Immunology, Medical research

## Abstract

Benefit-risk assessment (BRA) plays a vital role in the development and approval of drugs and biologics, including vaccines. The U.S. Food and Drug Administration recently published the final guidance “Benefit-Risk Assessment for New Drugs and Biological Products”, which presents a structured framework for evaluating the benefit-risk (BR) of drugs and biological products and encourages the use of quantitative BR analysis (qBRA) to support complex decision-making. Unique challenges arise when assessing the BR of prophylactic vaccines, including the extrapolation of clinical trial data to real-world outcomes. To address these challenges, qBRA has been used to assist in some cases of decision-making related to vaccines. This article explores key steps and considerations for the qBRA of vaccines within the FDA BR framework. We explore the processes of “Framing the Research Question and Scope,” “Identifying Key Benefit and Risk Endpoints,” “Selecting the Quantitative Approach and Characterization of Uncertainties,” “Gathering Data and Establishing qBRA Model Assumptions,” and “Interpreting and Communicating the BRA Results,” highlighting major considerations for each step. By carefully considering these processes and their challenges, we aim to help develop qBRAs that effectively inform decisions, ensuring that the benefits of authorized and licensed vaccines outweigh their risks.

## Introduction

**Fig. 1:**
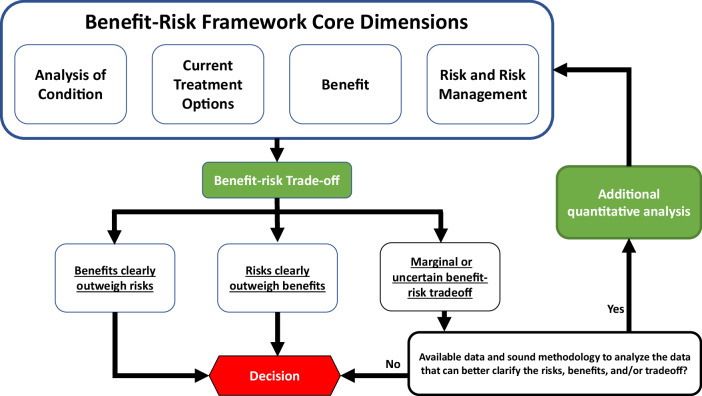
Decision tree for additional quantitative analysis in BR assessment for medical products. The upper panel illustrates the four core dimensions (Analysis of Condition, Current Treatment Options, Benefit, and Risk and Risk Management) that inform the primary benefit-risk trade-off assessment. Subsequent arrows indicate the flow of logic based on the clarity of this trade-off: clear outcomes (where benefits or risks clearly outweigh the other) lead directly to a final decision. In cases of marginal or uncertain trade-offs, the framework will include additional quantitative analyses if sufficient data and sound metholody exist, looping back to the core dimensions, otherwise a judgmental decision has to be made considering uncertainty. The red hexagon symbolizes the final regulatory or clinical decision point.

Under the fifth authorization of the Prescription Drug User Fee Act (PDUFA V, Fiscal Years 2013–2017) the FDA committed to significantly improve and formalize the “benefit-risk assessment (BRA)” process when reviewing new drugs and biologics^1^. The FDA published the final guidance “BRA for New Drugs and Biological Products” in 2024, which outlines a structured approach to assess the benefit-risk (BR) profile of drugs and biological products to assist regulatory decision-making^2^. FDA’s BR framework encompasses four key dimensions: Analysis of the Condition, Assessment of Current Treatment Options, Evaluation of Benefits, and Assessment of Risks and Risk Management. The FDA’s ultimate decisions on BR are carefully guided by a thorough consideration of available evidence, uncertainties, potential risk management strategies, and the necessary trade-offs between the benefits and risks. The BR framework serves as a foundation for a comprehensive, consistent, and transparent process to synthesize evidence and determine whether the benefits of the product outweigh the risks. The FDA guidance further states that additional quantitative BR analysis (qBRA) can be valuable to support structured BR assessment for complex decision problems; in essence, applying more comprehensive or advanced quantitative analysis to address critical and challenging questions for decision-making. When the benefits of a particular intervention clearly outweigh its risks, or vice versa, conducting a comprehensive qBRA in addition to the framework may not be necessary. However, in cases where marginal BR or considerable uncertainties make decisions challenging, additional qBRA can provide the necessary clarity (Fig. 1). A qBRA can include, but is not limited to, modeling real-world benefit and risk outcomes, integrating benefits, risks, and trade-offs in a combined analysis, analyzing the impact of uncertainty, or incorporating patient preference in the BR determination. The qBRA can facilitate discussion, assist in decision-making processes, and communicate BR determinations to the public.

Vaccination is a significant milestone in public health, effectively safeguarding countless lives worldwide by preventing infectious disease pandemics and epidemics. The rapid deployment of mRNA vaccines in the United States during COVID-19 pandemic through Emergency Use Authorization (EUA)^3^ and Biological Licensure Application (BLA)^4^ has been instrumental in averting millions of hospitalizations and deaths^5,6^ underscoring the critical role of safe and effective vaccines. The BRA is central for decision-making concerning vaccine development and market approval. Regulatory considerations for vaccines used to prevent infectious diseases differ from those for drugs and biologics used to treat existing medical conditions. Vaccines are generally administered to large populations without the disease, so the risk tolerance for vaccine use is generally lower than that for drugs and biologics intended for treatment. BRAs of vaccines also present unique challenges. To obtain sufficient statistical power to demonstrate vaccine efficacy typically requires very large numbers of trial participants, which takes time to recruit and is often unattainable during any crisis, e.g., pandemics, when a vaccine is urgently needed. Also, the vaccine efficacy and safety observed in clinical trials cannot directly translate into real-world benefits and risks. The effectiveness of vaccines in the real-world depends on many factors, such as population immunity, breadth and duration of the elicited immune response, individual disease susceptibility, intensity of exposure, reproductive rate of the disease-causing agent and circulating strains, most of which are constantly evolving. Vaccine effectiveness could vary by subpopulation, geographic region, climate^7^ and time, and could change due to other societal interventions. In addition, rare/very rare adverse reactions to a vaccine may not be observed in clinical trials due to limited sample size, selection of study population and relatively short follow-up, while they may emerge when the vaccine is deployed in a real-world setting. Also, the efficacy outcomes used in vaccine clinical trials generally include only medically attended disease, and rarely include more severe outcomes, such as hospitalizations and death, which are more relevant for BRA. These uncertainties pose significant challenges for the BRA of vaccines. Moreover, sometimes clinical trials rely on surrogate endpoints and immunobridging, which allows extrapolation of vaccine effectiveness by comparing immune response from approved uses to new indications, such as for different age groups, dosages, or co-administration with other vaccines. Immunobridging is also used to infer effectiveness in the accelerated approval of vaccines when needed for rapid deployment of vaccines in pandemic situations, or when conducting a full-scale clinical efficacy endpoints trial may not be feasible or fast enough to provide prompt protection to vulnerable populations. In such cases, the vaccine effectiveness must be confirmed, in some cases using real-world studies conducted post-authorization. Another distinctive aspect of the BR consideration for vaccines is the broader public health benefit they may provide, extending beyond the protection of the individual vaccine recipient, by potentially lowering disease transmission. Understanding and addressing these complexities is vital for the evaluation of the BR profile of vaccines to inform regulatory decisions.

Numerous published studies employing quantitative methods or modeling, as detailed in this work, have tackled specific questions about the BR balance of vaccines. Additionally, the FDA’s Center for Biologics Evaluation and Research (CBER) has leveraged qBRA to inform both EUAs and BLAs for COVID-19 vaccines. The publication of FDA BR guidance, our regulatory experience with COVID-19 vaccine during the pandemic and the broad public interest on outweighs prompted us to publish an article articulating the process of qBRA for prophylactic vaccines. In this article, we explore the key steps and considerations for the qBRA, which are aligned with based on our experience with COVID-19 vaccines and our review of published literature. The vaccine products, decision context, data, and conclusions discussed as examples in this paper may have evolved over time. Our aim is to establish good practices for qBRA of vaccines and illustrate the steps and considerations, rather than endorsing the conclusions of specific studies.

Even though we use “vaccine” as an abbreviated term throughout this paper, we focus only on prophylactic vaccines for the prevention of infectious diseases and not on individualized or therapeutic vaccines, such as cancer vaccines, which require distinct BRA considerations. Furthermore, the qBRAs discussed in this article do not include cost-benefit modeling.

## qBRA for vaccines: steps and considerations

**Fig. 2:**
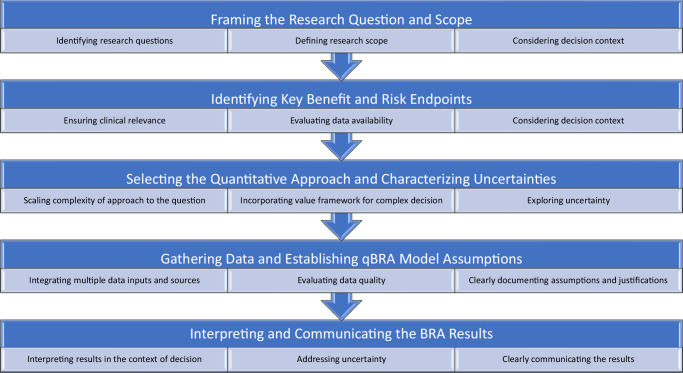
Steps and considerations for conducting qBRA for vaccines. The set of steps begins by clearly defining the research question and scope, ensuring alignment with stakeholders, and tailoring the analysis to the decision at hand. The next step involves identifying key benefit and risk endpoints that are clinically relevant and supported by reliable data. The appropriate quantitative approach is then selected, matching complexity to the question and incorporating techniques to address uncertainty. Data are usually gathered from various sources, with an emphasis on quality. Finally, results are interpreted in context, acknowledging uncertainties, and communicated clearly using visual tools for easy understanding by stakeholders.

This section outlines six key steps in conducting a qBRA for vaccines: framing the research question and scope, identifying key benefit and risk endpoints, selecting the quantitative approach and characterizing uncertainties, gathering data, and establishing qBRA model assumptions, and interpreting and communicating the BRA results (Fig. 2). Below, we discuss specific considerations for each step within the context of vaccine qBRA.

### Step 1: Framing the research question and scope

#### The quantitative BR assessment should begin by clearly defining the research question and scope, thereby ensuring alignment with stakeholders, and enabling a tailored analysis that directly addresses the decision at hand

Additional quantitative analyses have an important place within the BR framework (BRF) to help address outstanding decision-making questions. All BR assessments adhere to a BRF, starting with an examination of existing evidence and uncertainties across the BR dimensions. These dimensions include an analysis of the condition, current treatment options, benefits, risks, and risk management strategies. Subsequently, decisions are made after trade-offs based on the vaccine BR profile. For vaccines, the stakeholders vary based on the purpose of the analysis and medical product and may include regulators, public health decision-makers, healthcare professionals, and the public. When the BR balance is marginal, understanding stakeholder values and their respective trade-offs becomes more important. The qBRA methodologies can be employed to incorporate weighting of benefit and risk outcomes for trade-off using stakeholders’ preferences including those of the patients. Additionally, qBRA is often used to evaluate the impact of uncertainties and gain insights into the repercussions of remaining uncertainties concerning benefits, risks, or the overall BR trade-off.

The initial step in conducting a qBRA involves clearly defining the research question and outlining the analysis scope. This focuses the study on the critical question by establishing clear research objectives, which informs study design, data collection, and methodology selection. In addition, a well-defined research question establishes a common understanding between analysts and decision-makers on key issues and the approach to addressing them. This alignment ensures the analysis is relevant to the decision context and helps reach consensus within the team when interpreting the results. A robust research question possesses characteristics, such as specificity, feasibility, relevancy, and plausibility^8^. The research question should be clear and focused; an effective qBRA should be directly tied to the decision it seeks to inform. Many decisions are made over a vaccine lifecycle, including starting or halting a vaccine development, granting market approval, comparing a new vaccine to existing options, or developing public health policy. Additionally, qBRAs can be essential for evaluating long-term benefits and risks of the vaccine, vaccine deployment strategies, or the impact on the BR balance of new safety concerns emerging during the post-marketing phase.

Determining the scope of the study often involves identifying the target population, geographic region, and time period. This determination should consider the relevance of the decision context, the focus of the research question, the urgency of the decision, and the availability of relevant data. Based on our review of the literature, many qBRA focused on the overall population for which the vaccine is indicated^9–14^ while others focused on specific subpopulations with a relatively greater disease burden or a higher risk of adverse reactions related to vaccination^15–19^. Some qBRAs are conducted for use in specific countries or regions (e.g., high disease transmission vs. low disease transmission areas^20,21^. Some qBRAs have clearly defined analysis periods and focus on questions related to specific virus strains, vaccine products, vaccination programs or policies^20,22–33^. Below we provide several examples of qBRA studies to illustrate how research questions and study scopes were defined for specific decision contexts.

#### Comparing the BR balance of vaccine exposure to no vaccination

A central question often addressed by a qBRA is whether a vaccine’s benefits outweigh its risks compared to no vaccination. This is generally asked when current treatment options are limited and there are no alternative vaccines for the prevention of disease^9,10,12,15–19,21,29,30,33–54^. In 2021, FDA reviewed the BLA for the Pfizer-BioNTech COVID-19 Vaccine (CVmRNA) for individuals aged 16 years and older. This vaccine was recommended for use in persons aged 12 years and older in the United States under EUA. Real-world evidence since the EUA of mRNA COVID-19 vaccines, produced by Pfizer-BioNTech and Moderna, indicated that these vaccines were effective in preventing COVID-19 cases, hospitalizations, and deaths^55–57^. However, there were reports of elevated rates of myocarditis and pericarditis (i.e., inflammation of the heart or its lining) associated with mRNA COVID-19 vaccination, particularly among male adolescents and young adults^58^. A critical question the FDA faced at the time of CVmRNA BLA review was whether the benefits of the vaccine in preventing COVID-19 cases outweighed its myocarditis/pericarditis risks for male adolescents and young adults. This was the first BLA for a COVID-19 vaccine, and the impact of the risk of myocarditis and pericarditis on the overall BR balance of the mRNA COVID-19 vaccine was not clear at the time. The FDA conducted a qBRA to inform its BLA decision. FDA’s assessment^19^ involved comparing vaccination versus no vaccination for benefit outcomes, including the prevention of COVID-19 cases, hospitalizations, intensive care unit admissions (ICU), and deaths. In parallel, risk outcomes, including vaccine-attributable myocarditis and pericarditis cases, hospitalizations, and deaths were evaluated. Since the risks of myocarditis and pericarditis were highest among male adolescents and young adults, the FDA’s qBRA focused specifically on males aged 16–29 years old and limited the BR analysis to a period of 6 months post-vaccination based on the anticipated period of vaccine protection.

We also reviewed a list of publications on vaccine qBRA, focusing on the research questions being addressed^9,10,12,15–19,21,29,30,33–54^. One such example is a qBRA for recombinant zoster vaccine (RZV) conducted jointly by the Centers for Disease Control and Prevention (CDC), FDA^15^ and academic collaborators. The CDC’s Advisory Committee on Immunization Practices (ACIP) had recommended the use of RZV to prevent herpes zoster (HZ) and related complications in immunocompetent adults aged 50 years and older. However, a subsequent analysis of data from the Vaccine Safety Datalink (VSD), provided by US CDC, revealed a safety signal indicating a potential association between RZV and Guillain-Barré syndrome (GBS) in the target population^59^. Considering this emerging safety concern, a decision needed to be made regarding continuation of the RZV vaccination. A qBRA was conducted to provide insights into the benefits of vaccinating immunocompetent adults aged 50 years and older with the RZV compared to not vaccinating^15^. The key research question in this decision-making process was whether the benefits of RZV in preventing HZ outweighed the potential additional risk of GBS associated with RZV in the target population. The resulting analysis helped guide CDC’s decision to maintain its recommendations for vaccination^60^.

#### BR balance of a vaccine relative to other vaccines

When a new vaccine is introduced, sometimes there is another vaccine already approved for a similar (or the same) indication. If so, equipoise can often be assumed when the benefits and risks of the new vaccine are compared with existing vaccines, which help understand the additional benefits and risks associated with the new vaccine. Vaccines can be compared with alternatives from other brands, manufactured using different technologies, or with modified formulations or dosing schedules^23,32,33,47,61^. For influenza, the disease burden is significant among the very old, and the trivalent inactivated influenza vaccines (TIV) were initially recommended for this population. Subsequent studies reported significant hospitalization risks associated with influenza among healthy young children^62,63^. In response, in 2008, the CDC’s ACIP recommended routine annual vaccination of all children aged 6 months and older with TIV. However, clinical trial data had suggested that the efficacy of TIV in younger children might be lower than in adults. To address this unmet need, a live, attenuated influenza vaccine (LAIV) was developed in 2003. The FDA approved the use of LAIV for children aged 24–59 months in 2007, with warnings about an increased risk of wheezing and hospitalization, and exclusion of individuals with a history of recurrent wheezing or asthma from the indication. In this context, Oster et al. conducted a qBRA to compare the use of LAIV vs. TIV among the young population^47^. The study aimed to assess the incremental benefit of preventing influenza cases and the risk of medically significant wheezing and hospitalizations if the target group switched from using TIV to LAIV during an influenza season. The authors concluded that the overall BR balance of using LAIVs in young children was favorable, even though, in clinical practice, there may be inadvertent uses of LAIV in young children with high risk of wheezing or asthma.

As another example, Bollaerts et al.^32^ conducted a multi-criteria decision analysis (MCDA), a specific type of qBRA, to integrate the weighted benefits and risks of different pertussis vaccine formulations using data from pre-school children. Pertussis, caused by the bacterium *Bordetella pertussis*, is a common respiratory disease endemic worldwide. Pertussis vaccines have significantly reduced the burden of the disease^64^. Two main formulations were available for children at the time of analysis: whole-cell pertussis (wP) and acellular pertussis (aP) vaccines. The wP vaccine was introduced in Europe in the 1940s, while aP vaccines have been in use since the mid-1990s. Some evidence indicated aP vaccines have higher efficacy^65^. However, observational, and population-level studies suggested that introducing at least one dose of the wP vaccine into the routine immunization schedule provides better disease protection and longer-lasting immunity^65^. Nonetheless, the wP vaccine is associated with higher rates of reactogenicity and adverse events. The qBRA^32^ was conducted to assist decisions regarding the selection of pertussis vaccines—whether aP, wP, or a combination of both—by weighing their overall benefit against the risks using preferences from clinical and epidemiological experts. To understand the comparison between wP and aP, a technique called Monte Carlo simulation was used, which involves running many simulations with randomly sampled input values to generate a range of benefit and risk outcomes. The authors developed a scoring equation (BR score) that is a weighted average that combines a vaccine’s various benefits and risks into a single score ranging from 0 to 100, where a higher score indicates a better overall BR outcome based on the number of events prevented or caused and their perceived importance. Results of the simulations showed that wP generally had a better BR score than aP, although there was significant overlap in the range of likely BR scores of the two.

When safety concerns arise about a particular class of existing vaccines, a qBRA can help compare the BR balance of different vaccines within that class. For instance, Rotarix and RotaTeq are two vaccines designed to protect against rotavirus gastroenteritis (RVGE). Both vaccines have significantly reduced the incidence of pediatric diarrhea in the countries where they are used^66,67^. However, both have been associated with a slight increase in the risk of intussusception, a rare type of bowel obstruction. To better understand the comparative benefits and risks of the two vaccines, a qBRA^33^ was conducted. This assessment compared between Rotarix and RotaTeq on the number of prevented RVGE hospitalizations against the number of vaccine-induced hospitalizations due to intussusception.

#### Exploration of BR of alternative vaccination strategies

Various vaccination strategies are often considered to maximize the public health benefits, particularly when resources are limited. These strategies encompass decisions about who, when, and with what to vaccinate, as well as how vaccination can complement other public health interventions. The qBRAs are employed to evaluate the public health impact of different vaccination strategies^9,20–22,24,25,27,28,43,68–70^. In a study by Shiri et al.^9^, a qBRA assessed the BR ratio of two vaccine programs: one prioritizing booster doses for previously vaccinated populations to combat waning immunity, and the other prioritizing primary doses for unvaccinated populations to provide broader protection. Similarly, Liu et al.^69^ utilized a qBRA to assess the public health impact of various dosing interval strategies for COVID-19 vaccination in middle-income countries in Europe, considering constraints in COVID-19 vaccine supply. Their research aimed to determine if extending the interval between first and second COVID-19 vaccine doses could allow more people to receive primary vaccination, potentially generating greater overall public health benefit with limited vaccine supply.

### Step 2: Identifying key benefit and risk endpoints

#### It is imperative to identify key benefit and risk endpoints for qBRA that are clinically relevant, supported by reliable data, and relevant to decision context

Once the research question and scope are set up, the next step is to identify the key benefit and risk endpoints to be included in a qBRA. A value tree (also known as an attribute tree) can be used to map out key benefits and risks and help facilitate discussion. Some important points need to be considered when selecting key benefit and risk endpoints. The first is the clinical relevance of the study endpoints, including their ability to measure outcomes with the greatest impact on individuals or the overall public health, depending on the objectives. For licensure of a drug product, key benefit endpoints for a qBRA often align with clinical endpoints studied in the clinical trials to demonstrate the substantial evidence of effectiveness required for regulatory approval. While vaccine efficacy is typically used as the primary endpoint in a vaccine clinical trial, vaccine efficacy is not directly translated to the real-world vaccine benefit among the target population. The real-world benefits, such as number of cases or hospitalizations prevented, depend not only on vaccine efficacy but are also affected by other factors (e.g., disease incidence and severity among the target population). A vaccine may provide greater benefit in certain regions with higher disease transmission or among a population more susceptible to a disease or its complications, but less benefit in regions with lower disease transmission or population less susceptible to the disease or its complications. Furthermore, clinical trials often have strict inclusion criteria and controlled environments, which do not perfectly reflect the real-world conditions and diverse characteristics of the general population. The key benefit endpoints used in a qBRA for a vaccine are often actual measurable benefits. Examples are prevention of disease cases, hospitalizations and mortality; or reduction in severity and duration of the disease. Whether the data quality fits the purpose of the analysis is another important consideration. For example, FDA’s qBRA^19^ for Pfizer-BioNTech COVID-19 Vaccine BLA used prevented numbers of COVID-19 related cases, hospitalizations, ICU admissions, and deaths as the benefit endpoints. These endpoints were considered clinically important to individuals, as well as good measurements for public health impact. CDC COVID-19 Data Tracker has been collecting data on COVID-19 cases, hospitalizations, and deaths since the beginning of the pandemic, which provides good-quality data for the inputs of FDA’s qBRA.

Decision context is an important consideration when we identify key benefit and risk endpoints. A BR decision becomes challenging when an important risk is identified. In such situations, a qBRA is triggered to quantify such a risk and determine whether the benefits of a vaccine continue to outweigh its risks. FDA’s qBRAs for Pfizer-BioNTech COVID-19 Vaccine BLA^19^ and Moderna COVID-19 Vaccine BLA^18^ used vaccine-attributable myocarditis and pericarditis cases, related hospitalizations, and deaths as key risk endpoints representing the major safety concern at the time of the BLA review. CDC VSD and FDA Biologics Effectiveness and Safety (BEST) system were established to monitor adverse events post-vaccination. These data sources provided reasonably reliable data for FDA’s qBRA.

Key BR endpoints may also highlight unique aspects of the product fulfilling an unmet medical need. For example, in Canadian Aboriginal communities with a high prevalence of tuberculosis (TB), there is an urgent need for TB vaccination for neonates. However, there is a concern that many of these infants might have a higher risk of severe combined immunodeficiency (SCID), leading to increased risk of death due to disseminated Bacille Calmette-Guérin (BCG) infection post vaccination. Clark and Cameron employed a Markov model to simulate the progression of TB and SCID in children from birth to 14 years of age^16^. They explored how the BCG vaccine can fulfill unmet medical need of protection against TB among Canadian Aboriginal infants, and how this benefit needs to be balanced with the risk of SCID. The study’s findings suggested that the decision to administer the BCG vaccine is significantly influenced by the incidence of SCID in the population. If the risk of SCID is high, the potential harms of the vaccine might outweigh its benefits in preventing TB.

The endpoints measuring impact on the patient’s quality of life may also be considered in a qBRA if a unique benefit of the product is to improve the patient’s quality of life. Patient input may help identify the endpoints important to them. For example, a study by Lee et al.^43^ evaluated different pertussis vaccination strategies for healthy adolescents and/or adults. The quality-adjusted life-years (QALY) saved was included as one of the main benefit outcomes. The researchers obtained patient preferences for different health states from adults and parents of adolescents with confirmed pertussis disease, incorporating these preferences into a BR trade-off.

Patients who use drug products to treat diseases take certain risks in exchange for benefits from the drugs. A prophylactic vaccine, however, may help prevent the disease not only among vaccine recipients, but also potentially reduce transmission and extend the benefits to a larger population. Reduction of disease transmission brings a great public health benefit, including lowering the burden on the public health system (e.g., decreasing hospitalizations and emergency room visits), and minimizing social and economic disruptions (e.g., long-term shutdowns of schools or other community activities) often accompanying a pandemic. Particularly, the benefit of herd immunity among the general population may help protect pediatric and other subpopulations whose underlying medical conditions constitute contraindications to vaccination. Many qBRAs that inform decisions related to vaccination programs include reducing disease transmission as a key benefit endpoint. For example, the work by Hawkes and Good^71^ included reduction of COVID-19 transmission as one of key benefit endpoints when they examined the impact of childhood vaccination, which is in alignment with a goal of the NexGen COVID-19 vaccines^72^.

When considering key benefits and risks, it is important to consider not only short-term but also long-term vaccine benefits and risks, especially those related to severe and irreversible disease outcomes. Long-term adverse effects of vaccines can either be anticipated based on the class effect of similar vaccines, identified through long-term follow-up studies, or detected through post-marketing surveillance. A newly identified long-term effect may trigger a reevaluation of vaccine BR. In such cases, the newly identified vaccine benefit or risk should be included in a qBRA to determine if it changes the BR balance of the vaccine. The substantial long-term health problems encountered by COVID-19 patients (31–69% of patients according to some studies)^73^, known as long COVID, underscore the importance of including long-term effects in BR assessments of vaccines. This is exemplified by Shiri et al.’s qBRA^9^, which included long COVID when comparing the BR of booster vs. primary-only vaccinations of COVID-19 vaccines.

### Step 3: Selecting the quantitative approach and characterizing uncertainties

#### The quantitative approach should align with the complexity of the research question, address uncertainty, and incorporate value when needed

To calculate the number of individuals protected by a vaccine against a specific disease outcome (e.g., infection, hospitalization, or death), we multiply the outcome’s incidence rate in the unvaccinated population by the expected number of vaccine recipients, the vaccine’s effectiveness, and duration of protection. On the other hand, to calculate the number of vaccinated people who may experience an adverse event attributable to vaccination, the incremental rate of adverse events seen in the vaccinated population compared to the general population (background rate) is multiplied by the expected number of vaccine recipients. Quantitative approaches in qBRAs range from simple head-to-head calculations to sophisticated mathematical modeling. The approach depends on the particular BR question, the complexity of the issues, and data availability. In line with Occam’s Razor principle of parsimony, it is best to start with the simplest approach possible, reserving complex modeling for scenarios where it is truly necessary. It is important to remember that model simplicity does not imply inferiority, and limitations and uncertainties of analysis need to be fully discussed and considered in decision-making. Moreover, considerations, such as the quality and completeness of data on benefits and risks are often more important than the complexity of the analyses performed. The scientific literature provides a variety of quantitative methods tailored to specific research questions and challenges. Below, we explore some of the available approaches, including when and how to use them.

#### Simple head-to-head calculation of BR

When a quick decision needs to be made or data are limited, a quick side by side back-of-the-envelope calculation of identified key benefits and risks based on available information can help facilitate discussion and inform the decision. It also helps with transparency and communication with stakeholders. A widely used approach for simple head-to-head BR comparison is to use a set of common metrics for both benefit and risk. This approach enables a direct comparison of the vaccine’s potential benefits and risks. As an example, the FDA qBRA for Pfizer-BioNTech COVID-19 vaccine^19^ used disease cases, hospitalization, and death as a set of common metrics for both benefits and risks. Simple head-to-head calculations eliminate extensive assumptions about the weight of the benefit and risk endpoints, as well as the false precision implied by complex models. This approach is also suited for preliminary explorations of BR to decide whether a more comprehensive analysis is needed. While the common metrics for benefit and risk facilitate intuitive BR comparisons, it is essential to recognize that clinical interpretation of a metric could still differ, depending on the benefit and risk outcome in question. Using FDA’s qBRA^19^ as an example, the COVID-19 related hospitalizations prevented by the vaccine were considered to have much more severe consequences than hospitalizations due to myocarditis/pericarditis cases caused by vaccine. The former requires longer hospital stay and more intensive treatments, while the latter has a shorter duration, and is often intended simply to monitor the condition. It is important to account for the varying severity of the hospitalizations from COVID-19 and vaccine associated myocarditis/pericarditis when making a comparison.

Some other qBRAs used single combined BR metric like BR Ratio (BRR)^13,14,21,30,33,38,42,51,74^, BR Difference(BRD)^30,42^ or Net Benefit (NB), or paired BR matrices, such as Number Needed to Harm (NNH) and Number Needed to Prevent (NNP)^35^. The BRR is a ratio of prevented cases (benefit) over excess adverse events of interest (risk). The BRD and NB calculates the absolute difference between prevented cases (benefit) and excess adverse events associated with the vaccine (risk). While NNP is the number of people needed to vaccinate to prevent one case of a specific disease (benefit), NNH represents the number of people who need to receive a vaccination to observe a specific adverse event (risk). The common underlying assumption for these approaches is that prevention of disease and adverse reactions associated with vaccination are equally important. Therefore, uses of these endpoints may be misleading when the clinical importance of benefit and risk outcomes are significantly different.

#### Subgroup analyses

Disease risk, vaccine efficacy, and side effect profiles can vary significantly across different subpopulations based on factors like age, sex, health conditions or immune status. Subgroup analyses are often conducted in qBRA to better understand the BR profiles of different subpopulations. These results help determine appropriate vaccine target populations, enabling personalized decision-making, optimizing immunization programs, and promoting equity by identifying potential disparities in vaccine outcomes. As an example, Kitano et al.^30^ evaluated the benefits and risks of mRNA COVID-19 vaccines against the Omicron variant of SARS-CoV-2, stratifying the analysis by age, sex, and comorbidities. This stratification allowed the assessment of the expected benefits and risks of the BNT162b2 and mRNA-1273 vaccines across different demographic groups.

#### Incorporating value in qBRA

Typically, benefits and risks are different disease outcomes, making the trade-offs challenging. When the clinical importance of benefit and risk outcomes are significantly different, or a BR trade-off is preference-sensitive, incorporating value judgment is often needed. Some BR assessments translate benefits and risks into a single metric, such as Quality-Adjusted Life Years (QALYs), which combines the length of life (years) with a value reflecting health utility of different health conditions (0 and 1, respectively representing the worst and a perfect quality of life), estimated through surveys, interviews, including time trade-off interviews or models. The QALY approach is especially useful when patients’ quality of life is an important aspect of the BR consideration. For example, Kitano et al.^30^ employed Quality-Adjusted Life Years (QALYs) analysis to measure the benefits and risks of monovalent mRNA COVID-19 vaccines. They calculated the expected QALY gains per 100,000 vaccinees factoring in age, sex and comorbidities. This approach allowed for a comprehensive assessment of the vaccine’s impact on an individual’s quality of life, considering both the benefit of protection against COVID-19 and the risk of potential vaccine adverse effects.

A different approach, MCDA, incorporates multiple benefit and risk effects (decision criterion) and weights, producing BR performance scores for different vaccination alternatives. MCDA could be considered a special form of decision tree model that incorporates weights of decision criterion in a qBRA. To determine the weight for each criterion requires value judgment. The weights can be generated from preference studies of stakeholders, including patients, physicians, and other decision-makers. Alternatively, some studies^75^ used health utility to calculate the weights. Bollaerts et al.’s^32^ integrated qBRA approach shows how to combine MCDA with individual-level state transition modeling to analyze risks, benefits, values, and uncertainties. It also demonstrates how to conduct preference elicitation through a workshop using swing-weighting^76^ and then incorporate it into MCDA.

#### Analysis of Uncertainty

Some uncertainty is unavoidable at the time of a decision. It is important to understand the impact of uncertainty on the BR balance and take it into account when making the decision. The Monte Carlo simulation approach uses statistical distributions to incorporate input uncertainty and generate probabilities of outcomes through random sampling and model simulation. The uncertainty of outcomes from Monte Carlo simulation is represented by a probability distribution, providing decision-makers with an overall picture of possible outcomes to better inform decision-making. In their MCDA study for the BRA of pertussis vaccines, Bollaerts et al.^32^ used Monte Carlo simulations to assess the impact of data uncertainty on the overall BR scores. Model estimates of expected number of events for each outcome were presented with 95% confidence intervals.

To evaluate the impact of uncertainties and variations in the input parameters of a qBRA model, sensitivity and scenario analyses are also often conducted. Sensitivity analysis involves systematically varying a single input parameter across a range of uncertainties to assess its impact on the overall BR balance of the product. Sensitivity analysis can be used to find model inputs with the greatest impact on BR, and to identify the data gap and direct future efforts on data collection to improve the qBRA. For example, in study by Bollaerts et al.^32^, sensitivity analyses were performed to assess the impact of data uncertainty and changes in preference weights on the overall BR scores. Marcelon et al.^45^ conducted a qBRA of the quadrivalent HPV vaccine for preventing anal cancer in young adolescent boys. To ensure the robustness of their findings, they conducted sensitivity analysis to investigate how the weights assigned to specific criteria within their MCDA framework influenced the outcome of the BR model. Scenario analysis, another approach, involves simulating different hypothetical scenarios, which could be different decision options or combinations of different uncertainties, to explore their implications on BR. For combinations of different uncertainties, the scenarios could include the most likely, optimistic, and pessimistic potential values, providing insights into the model’s robustness and the impact of various combinations of uncertainties on the BR results. The studies by Funk et al.^19^ and Marcelon et al.^45^ show carefully designed scenarios and sensitivity analyses. The BRA conducted by Funk et al.^19^ assessed the benefits and risks of the COVID-19 vaccine across different pandemic scenarios, including a base scenario resembling the pandemic state at the time of analysis, and a most likely and a worst-case future pandemic scenario. These scenarios changed the COVID-19 incidence, vaccine efficacy, and vaccine-attributable myocarditis/pericarditis rates. Their results showed that the vaccine’s benefits were significantly greater when the COVID-19 incidence was high, but the overall benefit outweighed the risks across all scenarios.

#### Modeling Transmission of Infectious Diseases for BRA

The SIR-type compartmental (transmission) models are mathematical tools often used to simulate the spread of infectious diseases among the population. The basic SIR model divides the population into three compartments: S (Susceptible), individuals who can contract the disease; I (Infected), individuals who are currently infected and can transmit the disease; and R (Recovered), individuals who have recovered from the disease, assumed to be immune and not transmit the disease. People transit from one compartment to the next based on infection and recovery rates. A qBRA often employs an SIR-type model when the primary decision goal is to reduce disease transmission within the population. However, it is important to keep in mind that SIR-type models can be complex. The population’s susceptibility to the disease, the effectiveness of the vaccine to reduce viral shedding, human contact behavior, and the infectious agent’s attack rate influences the rate of infection. Furthermore, the SIR model developed for a particular situation may not necessarily apply to others. For example, to assess childhood COVID-19 vaccination strategies, Hawkes and Good^71^ employed a deterministic SIR model with 58 parameters, with a set of parameters for Australia and a different one for Canada. Their model incorporated vital dynamics of population (births, deaths, and age), social distancing intervention, vaccine efficacy, population susceptibility, human-to-human contact, and other factors. These models could be valuable tools, but they must be used with caution and awareness of limitations. Lastly, in addition to SIR-type models there are other types of useful disease/vaccine impact models, including agent-based models (ABM)^77^ and network-based models^10^.

### Step 4: Gathering data and establishing qBRA model assumptions

#### It is very important to meticulously gather data from diverse inputs and sources, rigorously evaluate data quality, and clearly document all assumptions and justifications

Reliable input data are fundamental prerequisites for robust qBRAs. This includes comprehensive data on vaccine efficacy/effectiveness, vaccine safety profiles, disease epidemiology, health care utilization and relevant population characteristics. Vaccine qBRAs often use a mixture of data sources, including clinical trials, observational studies, vaccine surveillance, regulatory and government reports, scientific literature, and expert consultation. Input data that meet fundamental elements of quality and integrity (ALCOA principles^78^) are basic prerequisites to perform robust qBRAs. As new data emerges, qBRAs must be revisited and potentially revised or recalibrated to ensure they reflect the most up-to-date knowledge.

#### Clinical Trial Data

Clinical trial data must be accurate, reliable, and fit-for-purpose, and that sponsors should use a risk-based approach to ensure data integrity, focusing quality control efforts on the most critical data points^79^. These rigorously controlled studies provide essential measures of vaccine efficacy, demonstrating its ability to prevent disease outcomes under the clinical trial’s conditions. Key considerations for quality evaluation of clinical trial data include study protocol and adherence, missing data, endpoint validation and adjudication, and consistency across site and time points^80–82^. It is important to understand the differences between the vaccine efficacy demonstrated by the clinical trials and real-world vaccine effectiveness, an important input for vaccine qBRA. Although the outcome is usually determined more rigorously in a trial, the inclusion criteria may limit its ability to represent the larger real-world population. Also, the sample sizes of clinical trials are usually insufficient to determine risks of rare adverse outcomes risk. Furthermore, clinical trials for vaccines aimed at preventing pandemic infectious diseases or targeting specific high-risk populations often face a challenge: directly measuring vaccine efficacy through disease endpoints can be impractical. As a result, surrogate endpoints, such as antibody titers, to assess the effectiveness of these vaccines, are frequently relied on. How these surrogate endpoints correlate with actual vaccine protection needs to be confirmed post-marketing^83^. Data collected during clinical trials establish the initial BR profile of the vaccine, allowing for continuing quantification of BR when it is used in real-world setting. For example, Oster et al.^40^ focused on assessing the benefits and risks associated with the use of a live-attenuated influenza vaccine in young children. Their study primarily relied on data from a large phase III clinical trial offering a baseline against which the vaccine’s BR profile could be continuously evaluated and refined post-marketing based on real-world evidence.

#### Real-World Evidence (RWE)

Vaccine qBRAs often use RWE derived from Real-world data (RWD) in addition to clinical trial data. RWE studies are important to confirm real-world vaccine effectiveness among diverse populations and against evolving pathogen strains^84^. Furthermore, the limited sample size and follow-up in the clinical trial likely hinder the observation of long-term vaccine effects and rare adverse events. RWD can include different data sources, such as vaccination registries, insurance claim data, and electronic health records. All the FDA guidance documents on real-world evidence (RWE) data quality consistently emphasize that for RWE to be considered reliable for regulatory decision-making, the underlying RWD must be “fit for use,” meaning it is sufficiently relevant and reliable to address the specific research question, with careful consideration given to data provenance, accuracy, standardization, temporal coverage, and any biases inherent to the source^85–88^. RWE can be generated from observational studies, externally controlled trials, or randomized controlled trials using RWD to capture clinical endpoints. By integrating RWE with clinical trial findings, we can gain a better understanding of a vaccine’s benefits and risks over time, especially after a larger-scale vaccine deployment. As an example, in 2009 FDA licensed a high-dose trivalent inactivated influenza vaccine based on serological data collected in the clinical trials. Subsequently, FDA conducted a retrospective cohort study using US Medicare data to confirm the real-world effectiveness of high-dose inactivated influenza vaccine. The results of the analysis confirmed the significantly greater effectiveness of the high-dose trivalent inactivated influenza vaccine compared to the standard-dose in preventing influenza-related medical encounters among individuals greater than 65 years old. Furthermore, using the large study population with health claims data, this study established evidence of a statistically significant reduction in influenza-related hospitalizations and deaths with the high-dose vaccine, outcomes not previously observed in randomized clinical trials^83,89^.

#### Vaccine Surveillance Systems

Surveillance systems track vaccine uptake and monitor disease incidence and potential vaccine side effects on a large scale, allowing quantification of a vaccine’s true benefits and risks. Key considerations for quality evaluation of surveillance data include reporting timeline and completeness, case definition consistency, geographic and demographic coverage, data integration and signal detection sensitivity^90–93^. Passive surveillance systems (e.g., the Vaccine Adverse Event Reporting System (VAERS) in the US) rely on healthcare providers, laboratories, and the public to voluntarily report health-related events, such as disease cases or adverse drug reactions, to public health authorities. They provide a less resource-intensive tools for safety signal detection, but carry important limitations, including potential underreporting and misreporting. Active surveillances (such as US FDA’s CBER BEST System^94^ and CDC’s VSD^95^) are proactive public health systems in which officials directly contact healthcare providers, laboratories, or the public to actively seek out information on specific health events or diseases. These systems provide more precise adverse event data for rate estimate; however, demand greater resources and more expertise^96,97^. While neither system captures data on every single individual in the US, they provide valuable information on vaccine safety in large and diverse populations. There are limitations even with active surveillance, potentially including lack of medical chart review for validation of causal associations, inaccuracy of the ICD-10-coded diagnoses, underrepresentation of uninsured populations, and participant bias in outreach-based data collection^96,97^. Understanding data limitations and considering them when interpreting qBRA results is critical to avoid bias that misinforms the BR decision. As an example, during a BLA review, an FDA team conducted a qBRA to evaluate the use of Moderna COVID-19 vaccine among males aged 18–64^18^. The team considered the evolving pandemic situation as well as the risk of myocarditis and pericarditis. The model leveraged data from the CDC’s nationwide Increasing Community Access to Testing (ICATT) program (for vaccine effectiveness), CDC COVID-NET (for US hospitalization data), and a meta-analysis of four health claims databases in the BEST System (for myocarditis and pericarditis rate, which was based on more than 8 million vaccine doses). The impact of uncertainty from these data were evaluated through sensitivity analyses and limitations were considered when interpreting results of the qBRA.

#### Published Literature and Meta-Analyses

Literature reviews are often conducted prior to qBRA. Peer-reviewed publications offer rich data on disease burden, effectiveness of vaccines, and potential side effects. When evaluating published literature, rigorous assessment of the quality of individual studies is critical. This involves examining study design, sources of bias and confounders, methods used to assess unmeasured confounders, and the appropriateness of statistical methods. Studies that follow published frameworks^98–101^ for study design and research reporting can provide high-quality evidence and model inputs for qBRA. When there are multiple studies on a specific outcome, meta-analyses can be used to systematically synthesize and generate mean estimates and standard deviations of outcomes for model inputs. Meta-analyses increase the statistical power and allow researchers to identify patterns or inconsistencies that might not be clear in a single study. For instance, Clark et al.^13^ compared the potential benefits and risks of alternative rotavirus vaccination schedules in low- and middle-income countries, specifically focusing on mortality reduction and the vaccine-associated risk of intussusception. They conducted a random-effects meta-analysis of data from published self-controlled case-series (SCCS) studies to calculate the pooled relative risks (RRs) of intussusception by dose and period, using them as inputs of the BR assessment of rotavirus vaccination. Notably, the strength of a meta-analysis relies on the quality of the included studies.

#### Collaboration and Data sharing

Collaborative efforts are important in data collection, particularly for RWD. The exchange of information, especially during a global pandemic, helps generate a larger body of evidence faster, align BR assessments, and foster rapid and consistent decision-making and public health messaging. In the United States, as a part of the FDA Sentinel Initiative (which is a national electronic system that uses real-world data from various healthcare sources to proactively monitor the safety of FDA-regulated medical products, including drugs, vaccines, and medical devices, after they have been approved and are on the market), FDA CBER developed the BEST System in partnership with other health organizations to expand capacity for evaluating biologics product safety and effectiveness that leverages high-quality data, analytics, and innovation to enhance surveillance, RWE generation, and clinical practice^94^. Similarly, the VSD is a collaborative project between CDC’s Immunization Safety Office, integrated healthcare organizations, and networks across the United States that collects information about vaccine administration and uses electronic health data from participating sites to monitor and analyze the safety of vaccines. Both FDA BEST and CDC VSD provided good sources of data for FDA qBRAs informing FDA regulatory approvals of COVID-19 vaccines. Furthermore, international data can be useful in vaccine qBRAs by providing a larger and more diverse dataset, allowing for the identification of rare adverse events, examining potential differences in safety and efficacy across populations, and informing decision-making in countries with limited local data. The importance of collaboration and global data sharing has been shown extensively during the COVID-19 pandemic. As an example, the FDA qBRA team for COVID-19 vaccine incorporated the United Kingdom Health Security Agency’s (UKHSA) surveillance data on Omicron vaccine effectiveness due to limited US data availability at the time of analysis.

The Accelerated Development of Vaccine BR Collaboration in Europe (ADVANCE) provides another example of a collaborative framework among stakeholders^102,103^. VAC4EU^103^, the successor of ADVANCE, with its extensive pan-European network, became a valuable resource for vaccine safety and effectiveness assessments. This platform is used to streamline qBRA processes, secure reliable data access, find data gaps, promote methodological innovation, and conduct collaborative proof-of-concept studies. It promotes the timely generation of robust evidence essential for informed decision-making within the scientific and medical communities in the European Union (EU).

#### Expert Input

Expert input can be invaluable in vaccine qBRAs, particularly when a rapid response is needed, data are limited, and outcomes are uncertain; or when societal values must be factored in. Expert panels, Delphi surveys, and formal elicitation methods provide insights from experienced clinicians, epidemiologists, and other subject matter experts. These experts could help interpret complex data, provide estimates of parameters in the absence of direct evidence, or gauge the severity and acceptability of potential side effects. The expert judgment is essential because quantitative models are often sensitive to input assumptions. The expert inputs ensure the qBRA reflect real-world clinical practice and the perspectives of those with extensive experience managing the disease and its consequences. The expert elicitation is illustrated by the previous example of qBRA performed by Bollaerts et al.^32^, to compare wP and aP vaccines in children. The authors conducted a preference elicitation workshop with four clinical and epidemiological experts and three observers from the ADVANCE consortium. Following International Society for Pharmacoeconomics and Outcomes Research (ISPOR) guidelines, the experts assigned preference weights to preventing pertussis and the risks of febrile convulsions, fever, hypotonic-hyporesponsive episodes, injection-site reactions, and persistent crying associated with vaccination. The resulting preference weights were used in a MCDA to compare the BR of wP and aP vaccines in children.

FDA, CDC, EMA, and the World Health Organization (WHO) all have advisory bodies providing expert consultation on challenging BR issues and BRA. The FDA Vaccines and Related Biological Products Advisory Committee (VRBPAC) focuses on vaccines and related biological products^104,105^. In the United States, CDC’s ACIP plays a key role in developing recommendations for vaccine use. The EMA Committee for Medicinal Products for Human Use (CHMP) plays a vital role in the authorization and approval of medicines, including vaccines for human use in the EU. The Strategic Advisory Group of Experts (SAGE) on Immunization serves as the WHO’s principal advisory group to provide expert advice and recommendations for matters related to vaccines and immunizations. The qBRAs are often presented in these advisory meetings to facilitate discussion.

#### Assumptions

When conducting qBRA, certain data gaps are inevitable. Examples include a lack of information on background disease rates, vaccine protection duration, effectiveness against new variants, existing population immunity, and rare vaccine side effects. Assumptions may need to be made in such cases. Importantly, the assumptions need to be explicit, properly justified, and clearly documented. It is vital to ground model inputs and assumptions on the best available evidence, ensuring they are scientifically plausible. A rigorous examination of every assumption is essential for supporting the integrity and trustworthiness of the BRA. Sensitivity analyses and scenario analyses are valuable tools for evaluating the impact of assumptions in vaccine BR models. As discussed in the previous section, by systematically varying model inputs, these techniques help identify the impact of uncertainty associated with assumption, assess a range of potential outcomes, and robustness of the models.

### Step 5: Interpreting and Communicating the BRA Results

#### The results should be interpreted within the appropriate context, inherent uncertainties should be acknowledged, and findings should be clearly communicated

As discussed in earlier sections, the qBRA supports the structured BR framework by addressing outstanding questions critical to decision-making. BR analysts and decision-makers typically work together to interpret the analysis’s results. Analysis results should be interpreted in the decision context, including consideration of disease condition, unmet medical needs, available treatments, overall benefit and risk evidence, risk management, trade-offs, and uncertainty. The interpretation of results should include a conclusion that answers the predefined research question and a discussion of its implications.

The findings of qBRA need to be communicated effectively to the stakeholders. Together with results and its interpretation, the input data, assumptions, and analytical approaches used should be clearly described. The validity, strengths, weaknesses, and sources of bias, as well as how these uncertainties may impact the BR conclusion should also be discussed. Transparency of qBRA helps build trust and influences the successful implementation of subsequent decisions. Various visualization tools used to facilitate the effective visualization and presentation of complex BRA have been published (see examples in Supplementary Information). For a deeper understanding of the uses of these tools in BRAs of medicinal products, we recommend exploring the work of the PROTECT BR group^106^, the CIOMS Working Group report on BR balance for medicinal products^107^ and examples and references listed in their appendix.

## Discussion

BRA establishes the basis for regulatory and public health decisions related to vaccines. The qBRA offers a tool to support structured BR assessment of vaccines. As depicted in Fig. 2, the process of a qBRA is multifaceted, starting with the identification of research questions and defining the scope, which involves consideration of the decision context and target population. Subsequently, key benefit and risk endpoints are determined while ensuring their clinical relevance and data availability. The selection of appropriate quantitative approaches should consider the complexity of the approach to match the research question and the use of methods to explore uncertainties inherent to the data and modeling. Data gathering and establishing qBRA model assumptions are also critical, and integration of multiple data sources, rigorous evaluation of data quality, and clear documentation of assumptions and justifications are essential. The final steps involve interpretation and clear communication of results in the context of the decision-making while addressing impact of uncertainty. By following these steps, qBRA provides a robust framework to address critical questions in supporting vaccine decision-making, facilitating the continuous refinement of vaccine BR profile as new evidence emerges.

## Supplementary information

Supplementary information

## Data Availability

No datasets were generated or analysed during the current study.
