# Supplementary material for: Quantitative benefit-risk analysis for prophylactic vaccines in the context of FDAs benefit-risk framework
Source: NPJ Vaccines. 2026 Feb 5;11:84. doi: 10.1038/s41541-025-01342-3 (PMC13083997; doi:10.1038/s41541-025-01342-3)
Supplement: Supplementary file 1 — Supplementary information [file 41541_2025_1342_MOESM1_ESM.pdf]

Supplementary For Quantitative Benefit Risk Analysis for Prophylactic Vaccines in the Context of FDAs Benefit Risk Framework

Hong Yang[a]*, Osman N. Yogurtcu[a], Ujwani Nukala[a], Patrick R. Funk[a], Hector S. Izurieta[b], Richard A. Forshee[a,c]

[a]Office of Biostatistics and Pharmacovigilance, Center for Biologics Evaluation and Research, US FDA, Silver Spring, MD, USA

[b]Office of Vaccines Research and Review, Center for Biologics Evaluation and Research, US FDA, Silver Spring, MD, USA

[c]Office of Surveillance and Epidemiology, Center for Drug Evaluation and Research, US FDA, Silver Spring, MD, USA

Various tools facilitate the effective visualization and presentation of complex BRA. Bar plots, for instance, are commonly used in benefit-risk assessments to visually compare the magnitudes of various benefits and risks associated with a medical intervention or treatment option. In a specific application, Funk et al. used bar plots (Figure 1A) to visually compare the number of prevented COVID-19 cases, hospitalizations, ICU admissions, and deaths with the number of vaccine-attributable myocarditis and pericarditis cases, hospitalizations, and deaths in 16-17 year-old males after vaccination, aiding in the assessment of the overall benefit-risk balance of the vaccine.[1]

Value trees aid in the discussion and prioritization of key benefits and risks, clarifying their relative importance. [1-3] As an example, Son et al.[4][2] (Figure 1B) used a value tree (also known as attribute tree) to communicate their COVID-19 vaccine benefit-risk assessment, illustrating how each criterion contributed to the overall BR score.

Effects tables offer a concise and transparent format for presenting key data relevant to a qBRA and has been recommended by EMA for use in regulatory submission. Particularly, Marcelon et al.[4] (Figure 1C) employed effects tables to summarize the ranges, extremes and observed measures for each BR endpoint in their qBRA of the quadrivalent HPV vaccine for use in young adolescent boys, with a focus on preventing anal cancer as a new indication.

In addition to their role in qBRA modelling (as discussed in the main text), decision tree or influence diagrams can be used to outline the factors contributed to a decision, describe analysis methodology, and map the relationship between model inputs and outputs. [5-7] For example; Kitano et al.[6] (Figure 1D) used a decision tree to present the inputs included in the benefits and risks calculations of HPV vaccination). The tree illustrates how pathways and outcomes associated with Human Papillomavirus (HPV) infection and vaccination were modelled.

It is essential to clearly communicate the results and inherent uncertainties that accompany the findings since the qBRA results are often based on the available data and the assumptions made at the time. A range of possible outcomes or the results from different model scenarios should be presented with likelihood of occurrence and appreciation of how those results influence confidence in the benefit-risk conclusion. For example. forest plots (e.g., Figure 1E) can be used to visually summarize results from qBRAs.

A summary table documents sources of input data, assumptions and justifications can help to ensure transparency of BRA (see for example tables in [1, 3, 8-11]). Yogurtcu et al.[3] (Figure 1F) used a summary table to present the model inputs of varying COVID-19 incidence rates, vaccine effectiveness against cases and hospitalizations, and myocarditis/pericarditis rates for six scenarios.

Additionally, heat maps are a promising tool for communicating patient-centric BRAs by succinctly visualizing individual patient outcomes (see Figure 1G, for example). The color-coded representation of treatment benefits and adverse events allows for rapid assessment of the risk-benefit balance at the patient level.

The qBRAs utilize various other visual tools, such as risk scales, pictograms, waterfall plots, difference displays, tornado diagrams, box plots, and dot plots. For a deeper understanding of the uses of visualization tools in BRAs of medicinal products, we recommend exploring the work of the PROTECT BR group[12],the CIOMS Working Group report on BR balance for medicinal products[13].

References

1. Funk PR, Yogurtcu ON, Forshee RA, Anderson SA, Marks PW, Yang H. Benefit-risk assessment of COVID-19 vaccine, mRNA (Comirnaty) for age 16–29 years. Vaccine. 2022;40(19):2781-9.
2. Son KH, Kwon SH, Na HJ, Baek Y, Kim I, Lee EK. Quantitative Benefit-Risk Assessment of COVID-19 Vaccines Using the Multi-Criteria Decision Analysis. Vaccines. 2022;10(12):13.
3. Yogurtcu ON, Funk PR, Forshee RA, Anderson SA, Marks PW, Yang H. Benefit-Risk Assessment of COVID-19 Vaccine, mRNA (MRNA-1273) for Males Age 18-64 Years. Vaccine: X. 2023.
4. Marcelon L, Verstraeten T, Dominiak-Felden G, Simondon F. Quantitative benefit–risk assessment by MCDA of the quadrivalent HPV vaccine for preventing anal cancer in males. Expert Review of Vaccines. 2016;15(1):139-48.
5. Phillips LD, Fasolo B, Zafiropoulous N, Eichler H-G, Ehmann F, Jekerle V, et al. Modelling the risk–benefit impact of H1N1 influenza vaccines. The European Journal of Public Health. 2013;23(4):674-8.
6. Kitano T. Stopping the HPV vaccine crisis in Japan: Quantifying the benefits and risks of HPV vaccination in quality-adjusted life-years for appropriate decision-making. J Infect Chemother. 2020;26(3):225-30.
7. Shiri T, Evans M, Talarico CA, Morgan AR, Mussad M, Buck PO, et al. The Population-Wide Risk-Benefit Profile of Extending the Primary COVID-19 Vaccine Course Compared with an mRNA Booster Dose Program. Vaccines. 2022;10(2).
8. Ledent E, Arlegui H, Buyse H, Basile P, Karkada N, Praet N, et al. Benefit versus risk assessment of rotavirus vaccination in France: a simulation and modeling analysis. BioDrugs. 2018;32:139-52.
9. Mayfield HJ, Lau CL, Sinclair JE, Brown SJ, Baird A, Litt J, et al. Designing an evidence-based Bayesian network for estimating the risk versus benefits of AstraZeneca COVID-19 vaccine. Vaccine. 2022;40(22):3072-84.
10. Lau CL, Mayfield HJ, Sinclair JE, Brown SJ, Waller M, Enjeti AK, et al. Risk-benefit analysis of the AstraZeneca COVID-19 vaccine in Australia using a Bayesian network modelling framework. Vaccine. 2021;39(51):7429-40.
11. Sinclair JE, Mayfield HJ, Short KR, Brown SJ, Puranik R, Mengersen K, et al. A Bayesian network analysis quantifying risks versus benefits of the Pfizer COVID-19 vaccine in Australia. npj Vaccines. 2022;7(1):93.
12. Hallgreen CE, Mt-Isa S, Lieftucht A, Phillips LD, Hughes D, Talbot S, et al. Literature review of visual representation of the results of benefit–risk assessments of medicinal products. pharmacoepidemiology and drug safety. 2016;25(3):238-50.
13. The CIOMS Working Group (WG) XII. Benefit-risk balance for medicinal products. 2023.
14. Neveu D, Mallett Moore T, Zambrano B, Chen A, Kürzinger M-L, Marcelon L, et al. Structured Benefit-Risk Assessment of a New Quadrivalent Meningococcal Conjugate Vaccine (MenACYW-TT) in Individuals Ages 12 Months and Older. Infectious Diseases and Therapy. 2023;12(10):2367-86.

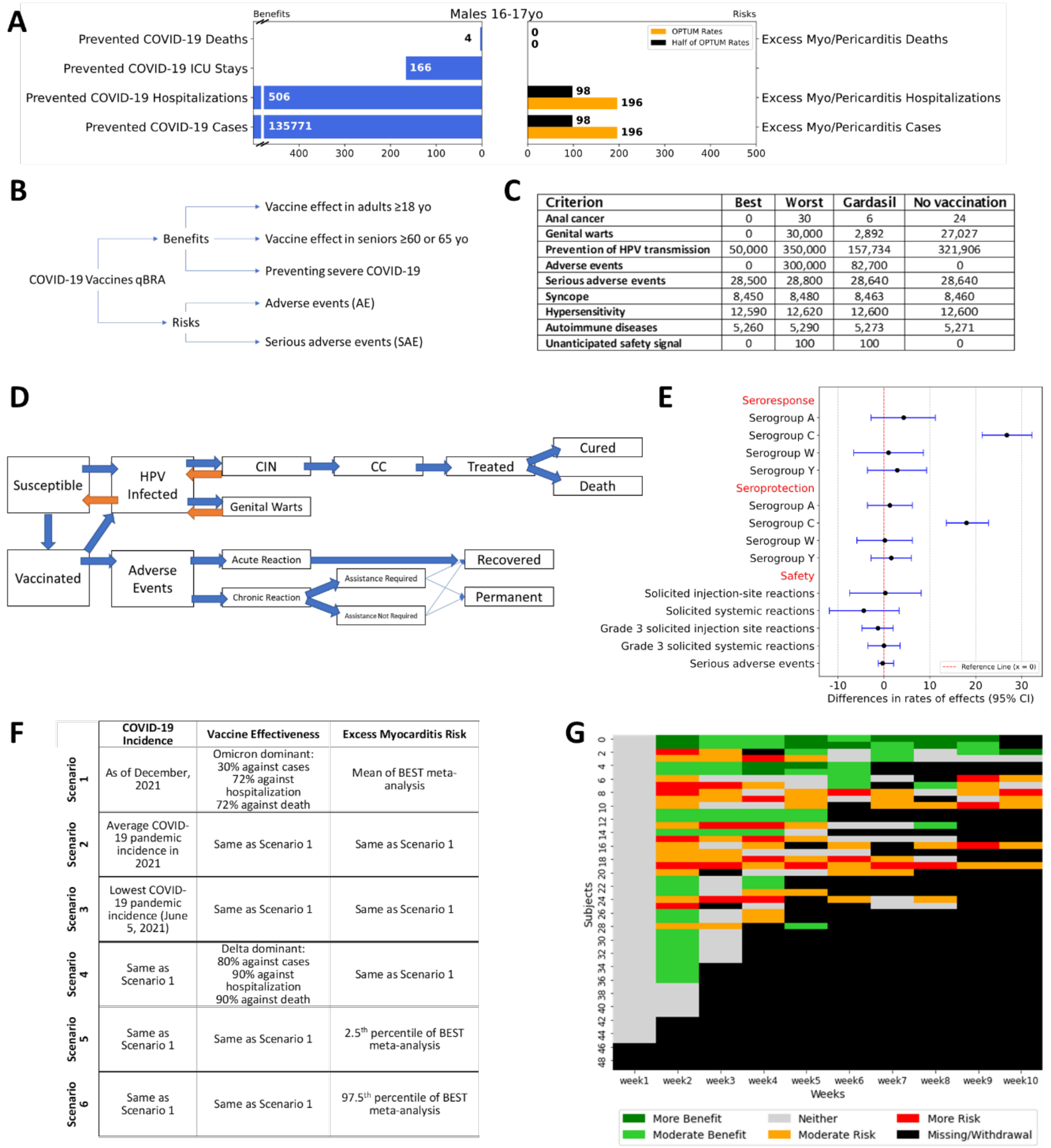

*Supplementary Figure 1: Tools for communication of a qBRA. (**A**) qBRA results of Scenario 2 (most likely) for the male 16-17 year-old population[1], part of the larger benefit-risk assessment of COVID-19 vaccine, mRNA (Comirnaty) for age groups 16–29 years. (**B**) Value (attribute) tree example adapted from[2], which was used to conduct a qBRA for COVID-19 vaccines using multi-criteria decision analysis. (**C**) Effects Table Example. Benefits and risks table for Gardasil in males (adapted from [4]). All numbers reported in units of per 100,000 persons over 30 years. (**D**) Decision tree model example from [6] was used to visualize the outcomes of the benefits and risks of receiving an HPV vaccine. CC, cervical cancer; CIN, cervical intraepithelial neoplasm. Initially, a susceptible individual who has not been exposed to HPV can either choose to become vaccinated or remain unvaccinated. If unvaccinated and subsequently infected with HPV, possible outcomes include the body's immune system successfully clearing the infection, the development of precancerous cervical changes, or the progression to cervical cancer. Additionally, HPV infection can also lead to genital warts. On the other hand, if an individual receives the HPV vaccine, there is a possibility of experiencing adverse events. (**E**) Forest plot showing a comparative analysis of MenACYW-TT (MenQuadfi®) and MCV4-TT (Nimenrix®) in a clinical study, focusing on meningococcal vaccine-naïve. The blue point estimate indicates the difference in benefit rates, with a positive value suggesting a higher benefit for MenACYW-TT. Conversely, the red point*

estimate represents the difference in risk rates, where a positive value implies a lower risk for MenACYW-TT. The confidence interval provides a range within which the true benefit or risk rate difference is likely to fall [14]*. The difference between the product of interest and comparator on each endpoint is represented by a horizontal line with a marker indicating effect size (e.g., odds ratio or risk ratio) along with error bars showing the confidence interval. A vertical line denotes no difference between the product of interest and comparators and divides the coordinate plane into two regions, favoring product of interest on the right and favoring comparator on the left. (**F**) Summary Table Example. Six model scenarios with varying COVID-19 incidence rates, vaccine effectiveness against cases and hospitalization, and myocarditis/pericarditis rates as described in*[3]*. (**G**) An example heat-map for a benefit risk comparison of a vaccine.*
